# MetalinksDB: a flexible and contextualizable resource of metabolite-protein interactions

**DOI:** 10.1093/bib/bbae347

**Published:** 2024-07-22

**Authors:** Elias Farr, Daniel Dimitrov, Christina Schmidt, Denes Turei, Sebastian Lobentanzer, Aurelien Dugourd, Julio Saez-Rodriguez

**Affiliations:** Heidelberg University, Faculty of Medicine, and Heidelberg University Hospital, Institute for Computational Biomedicine, Im Neuenheimer Feld 130.3, 69120, Heidelberg, Germany; Wellcome Sanger Institute, Wellcome Genome Campus, Cambridge CB10 1SA, United Kingdom; Heidelberg University, Faculty of Medicine, and Heidelberg University Hospital, Institute for Computational Biomedicine, Im Neuenheimer Feld 130.3, 69120, Heidelberg, Germany; Heidelberg University, Faculty of Medicine, and Heidelberg University Hospital, Institute for Computational Biomedicine, Im Neuenheimer Feld 130.3, 69120, Heidelberg, Germany; Heidelberg University, Faculty of Medicine, and Heidelberg University Hospital, Institute for Computational Biomedicine, Im Neuenheimer Feld 130.3, 69120, Heidelberg, Germany; Heidelberg University, Faculty of Medicine, and Heidelberg University Hospital, Institute for Computational Biomedicine, Im Neuenheimer Feld 130.3, 69120, Heidelberg, Germany; Heidelberg University, Faculty of Medicine, and Heidelberg University Hospital, Institute for Computational Biomedicine, Im Neuenheimer Feld 130.3, 69120, Heidelberg, Germany; EMBL European Bioinformatics Institute, Wellcome Genome Campus, Cambridge CB10 1SA, United Kingdom; Heidelberg University, Faculty of Medicine, and Heidelberg University Hospital, Institute for Computational Biomedicine, Im Neuenheimer Feld 130.3, 69120, Heidelberg, Germany; EMBL European Bioinformatics Institute, Wellcome Genome Campus, Cambridge CB10 1SA, United Kingdom

**Keywords:** single-cell, spatial, metabolomics, transcriptomics, cell–cell communication, database

## Abstract

From the catalytic breakdown of nutrients to signaling, interactions between metabolites and proteins play an essential role in cellular function. An important case is cell–cell communication, where metabolites, secreted into the microenvironment, initiate signaling cascades by binding to intra- or extracellular receptors of neighboring cells. Protein–protein cell–cell communication interactions are routinely predicted from transcriptomic data. However, inferring metabolite-mediated intercellular signaling remains challenging, partially due to the limited size of intercellular prior knowledge resources focused on metabolites. Here, we leverage knowledge-graph infrastructure to integrate generalistic metabolite-protein with curated metabolite-receptor resources to create MetalinksDB. MetalinksDB is an order of magnitude larger than existing metabolite-receptor resources and can be tailored to specific biological contexts, such as diseases, pathways, or tissue/cellular locations. We demonstrate MetalinksDB’s utility in identifying deregulated processes in renal cancer using multi-omics bulk data. Furthermore, we infer metabolite-driven intercellular signaling in acute kidney injury using spatial transcriptomics data. MetalinksDB is a comprehensive and customizable database of intercellular metabolite-protein interactions, accessible via a web interface (https://metalinks.omnipathdb.org/) and programmatically as a knowledge graph (https://github.com/biocypher/metalinks). We anticipate that by enabling diverse analyses tailored to specific biological contexts, MetalinksDB will facilitate the discovery of disease-relevant metabolite-mediated intercellular signaling processes.

## Graphical Abstract

**Graphical Abstract:**
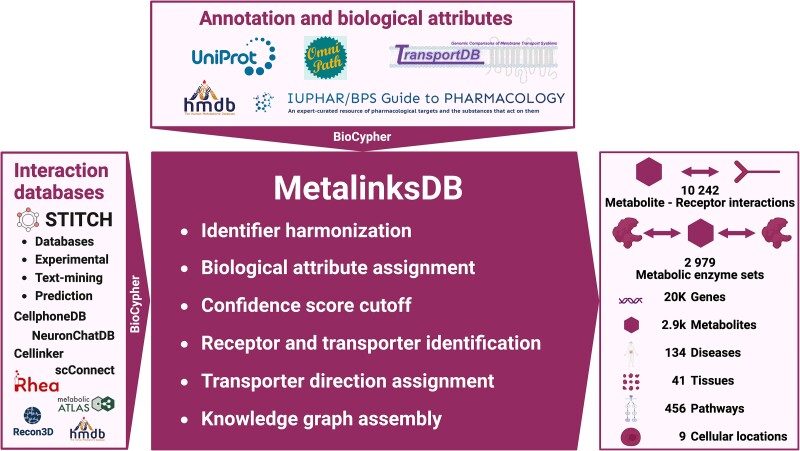

## Introduction

Metabolite-protein interactions are at the center of many cellular functions. The enzymatic catalysis of metabolites, and hence the use of nutrients, is crucial for cellular survival. Metabolites also act as signaling molecules that regulate enzymatic activity, energy homeostasis, and signaling [1, 2]. Metabolite-mediated signaling includes not only intracellular but also intercellular cell–cell communication (CCC). In recent years, technological advances in high-throughput sequencing, such as single-cell and spatial transcriptomics, have made the computational inference of protein-mediated CCC a standard practice [3, 4]. The most common approach relies on the co-expression between protein-coding genes, contextualized to CCC using prior knowledge [5]. As such, extensive effort has been dedicated to curating [6–8] and gathering resources focused on protein-mediated CCC [9]. Yet, this neglects a large portion of CCC interactions, such as the binding of extracellular metabolites to receptor proteins [2, 10–13].

Common sources for metabolite-protein interactions are the STITCH database [14], which consists of over 20 000 000 small-molecule protein interactions from various sources, the ‘IUPHAR Guide to Pharmacology’ resource [15], Rhea [16], and the Human Metabolome Database (HMDB) [17]. Moreover, information on metabolic enzymes, transporters, and their substrates can be found in genome-scale metabolic models like Recon3D [18] or the human metabolic atlas [19]. Yet, these generalistic databases do not necessarily focus on intercellular signaling.

Recent work, such as NeuronChat [20] and CellPhoneDB [21, 22], has focused on literature-curated knowledge in the context of metabolite-mediated CCC, while others [23–25] gathered subsets of interactions from text-mining or existing generalistic databases [15, 17] (Supplementary Table S1). As such, these resources are of limited coverage, typically lack transparency in their assembly process, focus on specific biological niches, and tend to remain outdated to newer versions of the underlying databases.

By assembling a knowledge graph using flexible BioCypher [26] adapters, we integrated all of the above resources into a comprehensive, versatile, and open-source database—MetalinksDB. The usage of such flexible adapters enables the seamless integration of new data sources as they emerge. Additionally, this modular approach further enables MetalinksDB to be easily utilized in other resources. As it stands, MetalinksDB is an order of magnitude larger than existing resources and offers biological information about pathways, diseases, and tissues, among others. Moreover, MetalinksDB enables metabolite-protein knowledge to be customized according to the quality or source of interactions. Additionally, MetalinksDB can be constrained to specific biological contexts—e.g. by filtering any interactions that are not relevant to a specific biological question. To enable users to make contextualization queries themselves, we assembled an interactive webpage (https://metalinks.omnipathdb.org/). We also provide programmatic access to MetalinksDB as a Neo4j knowledge graph (https://github.com/biocypher/metalinks) and via LIANA+—an all-in-one framework for CCC inference [27]. Here, we use MetalinksDB to analyze metabolite-protein interactions on a multi-omic data set of clear cell Renal Cell Carcinoma (ccRCC) [28]. Moreover, we combine MetalinksDB with LIANA+ [27] and showcase an application using a spatial transcriptomics dataset of acute kidney injury (AKI) [29].

## Results

### MetalinksDB: transparent and reproducible integration of interaction resources

The prior knowledge currently available for metabolite-mediated CCC is limited in terms of size, reproducibility, and extensibility, while also further lacking the possibility of customization. To address these limitations, we used BioCypher—a knowledge graph assembly framework that allows the straightforward incorporation of new resources and the continuous updates of existing ones upon changes [26].

**Figure 1:**
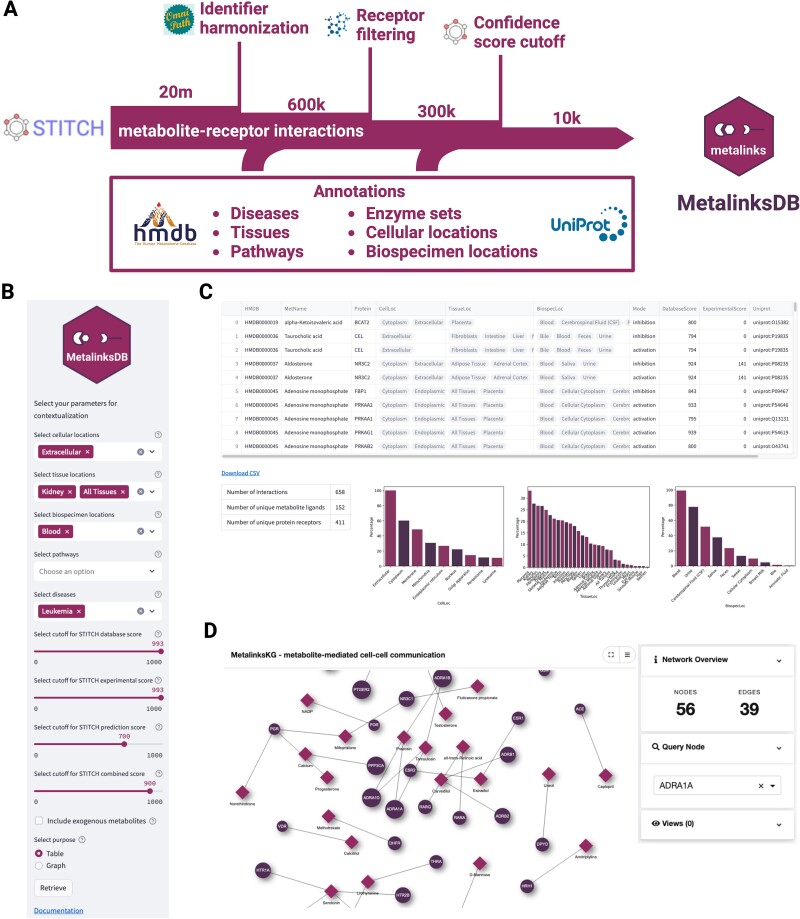
MetalinksDB graph assembly. (A) Filtering and annotation procedure during graph assembly. Over 20 million chemical-protein interactions from STITCH were filtered to the correct identifiers, receptor identity, and confidence. Moreover, annotations from several databases were added to the remaining metabolite-protein interactions. (B) Input panel for the web interface: the database can be queried for metabolites annotated to be present in a certain cellular location, tissue, biospecimen, pathways, or disease. The bottom panel enables the cutoff for the STITCH values to be chosen, as well as the desired output. (C) Contextualization table output panel: a table is generated from a user-defined query, while in the lower panel, several control plots are shown. The HMDB and Uniprot entries in the table are clickable hyperlinks that redirect to the respective HMDB and Uniprot web pages. (D) Graph investigation output panel: a graph is visualized with metabolites as diamonds and proteins as circles through the drugst. One interface [30], enabling the investigation of hubs and specific interactions.

By writing BioCypher adapters—short pieces of code that reproducibly format an input dataset—we integrated several prior knowledge resources, including STITCH [14], HMDB [17], Recon3D [18], the human metabolic atlas [19], Rhea [16], and several curated metabolite-receptor databases [20–24] (see Supplementary Notes S1 and S2). After several filtering steps and leveraging multiple annotation databases (Supplementary Fig. S1, Supplementary Table S2), we obtained a high-quality knowledge graph comprising ~10 000 metabolite-receptor interactions as well as the metabolic enzyme sets for over 2900 metabolites (Fig. 1A, Methods). Moreover, we added biological descriptors, such as diseases, pathways, and tissue locations to the nodes (metabolites and proteins), among others, for the edges (interactions) of the knowledge graph, allowing its contextualization to specific biological questions. Since the annotations of metabolites can be sparse, we wanted to investigate whether the coverage of biological descriptors is sufficient to enable the analysis with prior knowledge contextualized to specific conditions. We therefore quantified how many metabolites are annotated to a certain disease, tissue, cellular location, or pathway. We saw that most annotations lie in the range of 2%–20%, demonstrating that even with contextualized prior knowledge, the remaining coverage contains sufficient interactions (Supplementary Fig. S2).

To simplify the contextualization process, we deployed a web interface available at https://metalinks.omnipathdb.org (Fig. 1B–D). This interface has two main functionalities: (i) the contextualization of the MetalinksDB knowledge graph through interactive queries and (ii) the investigation of interactions of specific metabolites or proteins of interest. Both functionalities are accessed through a side panel that allows the input of several biological parameters such as tissue, cellular location, and biospecimen (Fig. 1B). The output table and several control metrics are displayed in the main panel, a download button allows the query results to be saved as a comma-separated file (Fig. 1C). In a second tab, specific metabolites or proteins can be investigated by visualizing the query results as interactive graphs [30] (Fig. 1D).

### MetalinksDB: a comprehensive and customizable knowledge graph

To assess the effectiveness and comprehensiveness of our database, we conducted a comparative analysis with existing databases (Supplementary Table S1). This comparison was carried out by dividing the interactions into two categories: metabolite-receptor interactions and metabolic enzyme sets (sets of enzymes associated with each metabolite).

**Figure 2:**
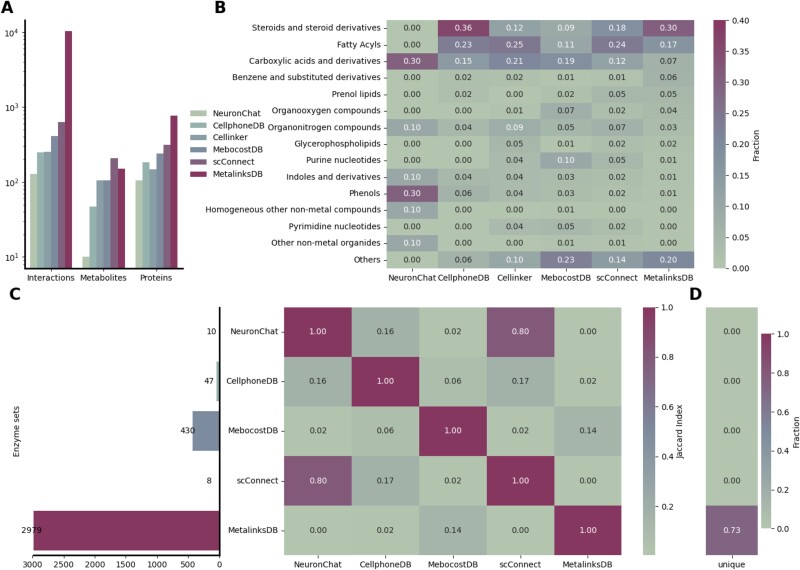
Comparison of MetalinksDB to other databases. (A) Comparison of metabolite-receptor resources. MetalinksDB contains a magnitude more interactions and the highest amount of proteins, while scConnect has the most metabolites. (B) A comparison of metabolite classes shows, e.g. that while CellPhoneDB and MetalinksDB contain a higher fraction of steroids than the remainder of the databases, MEBOCOST, scConnect, and Cellinker have a higher fraction of carboxylic acids. (C) Comparison of metabolic enzyme set size and overlap of enzyme sets. MetalinksDB has by far the highest number of metabolic enzyme sets associated with each metabolite. The Jaccard index heatmap shows that there is a high overlap between scConnect and NeuronChat. The highest overlap of MetalinksDB is with MebocostDB (0.17). (D) Fraction of unique metabolic enzyme sets between the databases highlighting that only MetalinksDB contains unique sets (0.69).

For the metabolite-receptor interactions, we quantified the number of connections, ligands, and receptors involved. MetalinksDB integrates multiple databases; as such, it encompasses a metabolite-receptor set that is an order of magnitude larger compared to other individual databases (~10 000 interactions in MetalinksDB versus fewer than 1000 in each database; Fig. 2A). Likewise, MetalinksDB comprises the highest number of proteins, and the second highest number of metabolites after scConnect (Fig. 2A), which we noticed to include metabolites that are not labeled as ‘extracellular’ in HMDB.

To gain insight into the composition of these databases, we further investigated whether there are specific classes of ligands that are more prevalent in different databases (Fig. 2B, Supplementary Fig. S3). We saw that MetalinksDB has the second highest fraction of steroids (0.30–0.00, 0.36, 0.12, 0.09, 0.18) while having similar fractions of fatty acyls (0.17–0.00, 0.23, 0.25, 0.11, 0.24) with NeuronChat, CellphoneDBv5, Cellinker, MebocostDB, and scConnect, respectively. MetalinksDB, however, has the smallest fractions of carboxylic acids (0.07–0.30, 0.15, 0.21, 0.19, 0.12) (Fig. 2B). Noteworthy, the database sizes differ by an order of magnitude, suggesting that while carboxylic acids make up a smaller fraction of MetalinksDB, our database still contains higher numbers from those (Supplementary Fig. S3D).

Next, we compared the size of the metabolic enzyme sets available in different databases. Through this comparison, we observed that MetalinksDB encompasses over five times as many metabolic enzyme sets as other databases (Fig. 2C). Furthermore, we saw that all metabolic enzyme sets present in other databases were also found within MetalinksDB (Fig. 2C). Moreover, MetalinksDB contained metabolic enzyme sets that were not found in the other databases (Fig. 2D). This expansion in enzyme coverage further strengthens the comprehensiveness of MetalinksDB as a resource for estimating metabolite abundance.

### MetalinksDB enables prior knowledge contextualization to specific disease contexts

**Figure 3:**
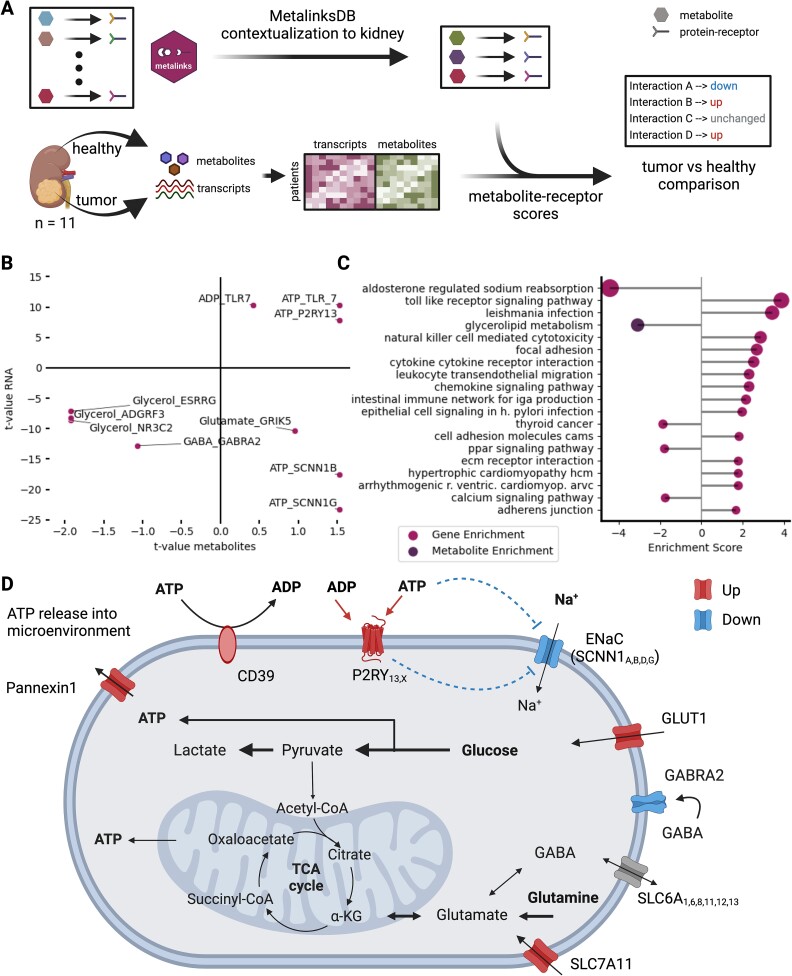
Bulk case study with contextualization. (A) Experimental strategy to infer cross-condition communication analysis using transcriptomic and metabolomic data of renal cancer patients. MetalinksDB was contextualized to metabolites present extracellularly in the kidney, blood, or urine. Differential abundance analysis was performed on each omics modality independently and the differential statistics (*t*-values) were used together with interactions from MetalinksDB to get the communication scores. (B) Top 10 deregulated interactions shown by the *t*-value of the transcriptome and metabolome differential abundance analysis. While in the upper right quadrant, many ATP and ADP interactions are displayed, the lower right quadrant shows interactions with ATP and ENac subunits (SCNN1_B,G_). (C) Deregulated pathways of metabolite and transcript pathway enrichment using univariate linear models. The highest enriched transcriptome interaction is ‘aldosterone-regulated sodium reabsorption’, while the highest enriched metabolite pathway is glycerolipid metabolism. (D) Graphical overview of the biological hypotheses generated using MetalinksDB. ATP, produced by enhanced glycolysis and enhanced ATP intake through upregulated GLUT1 transporters, is potentially released via the transporter Pannexin 1, which was found upregulated in tumor cells. In turn, in the extracellular space, ATP potentially activates purinergic receptors, such as P2RY13, while inhibiting sodium transporters, such as SCNN1 components.

To showcase the extended coverage of MetalinksDB and the possibility of contextualization, we examined the database’s capacity to identify metabolite-mediated CCC events and reproduce findings from the literature. Metabolite-mediated CCC plays an important role in several kidney diseases, including kidney cancer, which exhibits considerable metabolic dysregulation [31]. Hence, we used MetalinksDB to analyze a combined metabolomic and transcriptomics dataset of ccRCC—a specific form of kidney cancer. To decrease the number of putative metabolite-receptor interactions, and hence potential false positives, we filtered the MetalinksDB resource to metabolites that are annotated as present in the kidney, blood, or urine in HMDB and known to be extracellular (Fig. 3A, Supplementary Table S2). This contextualization of MetalinksDB reduced the metabolite-receptor resource to 3863 metabolite-protein interactions relevant to our specific context of kidney cancer (Supplementary Table S2).

The ccRCC dataset consists of metabolic and transcriptomic data from healthy and tumor samples of 11 kidney cancer patients [28] (Fig. 3A). Following a differential contrast between healthy and tumor samples using limma [32], we combined the contextualized metabolite-receptor knowledge from MetalinksDB with differential statistics (*t*-values) of both metabolite and gene expression (see Methods). This allowed us to obtain communication scores for putative metabolite-receptor interactions (Fig. 3B; Supplementary Table S3). In the top 10 metabolite-receptor interactions, we found multiple potential CCC events involving Adenosine Triphosphate (ATP) and Adenosine Diphosphate (ADP) (Fig. 3B), including ATP-P2RY13. This is in line with the known upregulation of anaerobic glycolysis in ccRCC—an important source for producing ATP [33, 34], which acts as an energy source and a signaling molecule [35–37]. Interestingly, the ATP/ADP nucleotide precursor adenosine was downregulated in tumors compared to healthy tissue, and we found it to be potentially interacting with the Adenosine A2b Receptor (ADORA2B; mean *t*-value = −1.34) (Supplementary Table S3). This interaction was also highlighted in the original publication [28].

As the next step, we performed pathway enrichment analysis using KEGG pathways [38] on the metabolite-receptor interaction scores, obtained using MetalinksDB knowledge. We found ‘aldosterone-regulated sodium reabsorption’ and ‘Toll-like receptor signaling pathway’ among the top dysregulated pathways (Fig. 3C). Both of these pathways contain receptors that are potentially bound (TLR7), activated (TLR3), or inhibited (SCNN1B, SCNN1G) by ATP (see ATP-TLR7, ATP-SCNN1B, and ATP-SCNN1G in Fig. 3). All of these are known to play a role in kidney physiology [39–41] or ccRCC [42]. For example, SCNN1B and SCNN1G are both part of the epithelial sodium transporter ENaC [43], known markers of ccRCC [44], and are implicated with sodium wasting in a SCNN1B genetic condition [45]. In line with this, the pathway ‘aldosterone-regulated sodium reabsorption’ was downregulated in the pathway analysis (Fig. 3C). Lastly, we found the interaction of ATP and the purinergic receptor P2RY13 (Fig. 3B), which is part of a receptor class known to be activated by ATP and ADP among other purines and pyrimidines [46–48]. Connecting those metabolite-receptor interactions, we hypothesized that the increased levels of ATP (Log2FC 2.33, uncorrected p.val 0.14; p.adj = 0.33) in tumor cells, likely originate from the increased anaerobic glycolysis. We further hypothesize that ATP could be released via the transporter Pannexin 1, which was also upregulated in tumor cells (Log2FC 1.00, p.adj 2.76 × 10^−4^) and in turn activating purinergic receptors, such as P2RY13 (Fig. 3B and D), while further inhibiting sodium transporters, such as SCNN1 components (Fig. 3B and D). The latter might in turn lead to diminished sodium levels in ccRCC, which has been previously proposed to serve as a prognostic and predictive factor of metastatic ccRCC [49] (Fig. 3D). Together, this analysis showcases MetalinkDB’s utility in identifying disease-specific, coherent metabolite-receptor interactions for hypothesis generation—insights that were consistent across different quality cutoffs of MetalinksDB (Supplementary Fig. S5) but could not be found with other databases—given their low coverage of kidney-specific metabolite-protein interactions (Supplementary Fig. S4).

### Using MetalinksDB to infer metabolite-mediated interactions driving kidney injury

**Figure 4:**
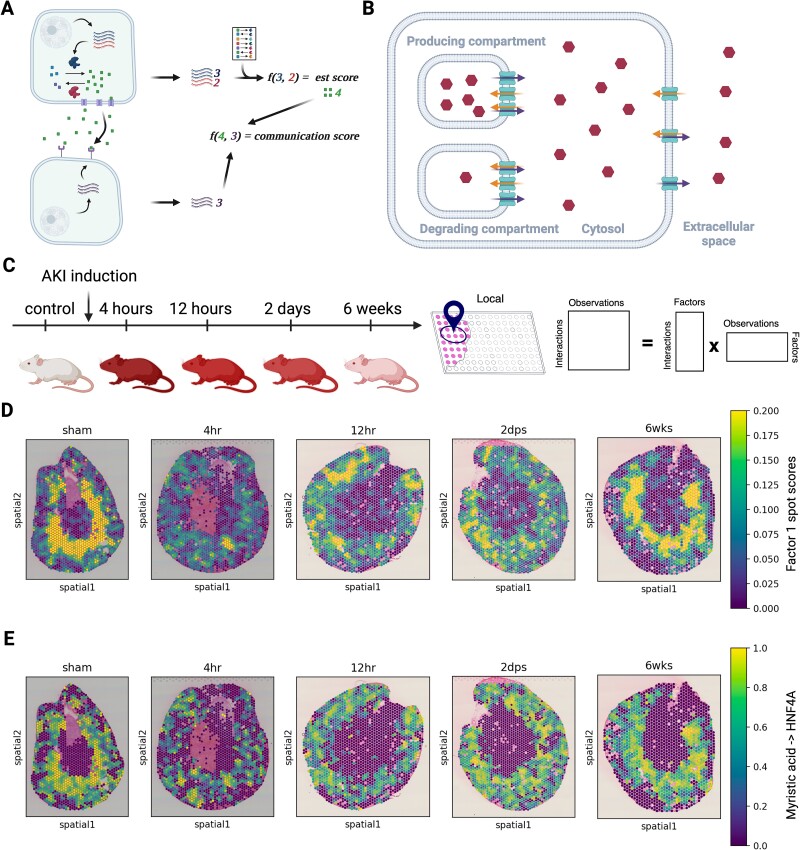
Metabolite-mediated CCC inference in AKI using LIANA+. (A) Principle of metabolite-mediated CCC inference from transcriptomics. (B) Visualization of importers and exporters. (C) Experimental setup to study murine kidney injury. AKI was induced in mice and samples for spatial sequencing were taken at 4 and 12 hours as well as 2 days and 6 weeks after treatment. (D) Factor 1 spot scores in mouse acute kidney injury spatial transcriptomics. The factor describes interactions that are strongly present in the control (sham) and is seen to disappear during injury, increasing back to sham levels during recovery. (E) Loadings of Myristic acid (HMDB0000806) -> HNF4A interactions, one of the top interactions comprising factor 1 (D). As in (D), the interaction is strong in the sham, absent after AKI, and reappears during recovery after injury.

Due to the absence of comprehensive single-cell metabolomic datasets, recent metabolite-mediated CCC tools infer metabolite abundance from transcriptomic data [20, 21, 25] and subsequently estimate communication scores between predicted metabolites and their known receptors; thus, generating hypotheses of putative metabolite-receptor interactions (Fig. 4A). Both steps require extensive prior knowledge, which is available in MetalinksDB (Fig. 4A).

One of the challenges in the context of metabolite estimation from transcriptomic data is that for a CCC event to take place, the metabolites have to be secreted in the extracellular space, and sometimes transported inside the receiver cell, to bind to the intended receptor (Supplementary Note S1). While ions and lipophilic substances can diffuse through membranes and vesicular transport systems, most molecules are transported via uni- or bidirectional protein transporters. To account for this, we collected information on importers and exporters from Recon3D [18], Human Metabolic Atlas [19], and TransportDB [50] and calculated a weighted mean to assess their presence, keeping metabolites with net positive import or export estimates (Fig. 4B, Methods).

To showcase the potential of MetalinksDB, we combined it with LIANA+ to infer CCC from spatial transcriptomics [27]. To increase computational efficiency and minimize false positive interactions, we constrained MetalinksDB to interactions, the metabolites of which were previously reported in the kidney. Along with the metabolites, we also retrieved their corresponding receptors and production/degradation enzyme sets. We used the enzyme sets to predict the presence of metabolites (Supplementary Fig. S6), along with providing MetalinksDB metabolite-receptor knowledge to LIANA+ (Methods). Using spatially-weighted Cosine similarity from LIANA+, we estimated metabolite-receptor interactions for each spot across five 10X Visium slides of murine kidney, acquired before and following AKI (Fig. 4C). We then used non-negative matrix factorization and identified three CCC patterns (factors), representing potential intercellular communication patterns (Fig. 4C, Methods). All three factors, especially Factor 1 and Factor 2, showed a strong decrease of CCC events with disease onset and an increase during recovery (Fig. 4D, Supplementary Fig. S7). Most prominently, Factor 1 showed interactions of fatty acyls like linoleic, myristic, and dodecanoic acid with the hepatocyte nuclear factor 4 alpha (HNF4A) (Supplementary Fig. S8), which were disrupted in the early hours following AKI, and subsequently seen to be recovered in the late time points (Fig. 4D and E; Supplementary Fig. S9). These CCC events were further characterized by the colocalization of fatty acyl and mRNA in the medulla of the healthy and recovered kidneys (Supplementary Fig. S9). This hypothesis is supported by the binding of HNF4A to several different lipids than the ones observed (linoleic acid -> HNF4A, not in MetalinksDB but suggested in the literature) [51], and HNF4A signaling was found to drive recovery after AKI in mice [52]. Thus, here we show that combining MetalinksDB with LIANA+ facilitates the formulation of robust hypotheses of metabolite-mediated CCC relevant to specific disease contexts (Supplementary Fig. S10).

## Discussion

In this paper, we report the assembly of a database called MetalinksDB, comprising the interactions between metabolite ligands and protein receptors as well as metabolic enzymes that produce and degrade these metabolites. Not only is MetalinksDB, to our knowledge, the most comprehensive database of its kind, but it is also the only one that is customizable through its flexible infrastructure.

To make this database easily accessible, along with flexible programmatic access, we built a web interface to allow users with no computational experience to customize, investigate, and download the database.

We demonstrate the application of MetalinksDB in analyzing both bulk and spatial transcriptomics data. From bulk transcriptomic and metabolomic data, we find interactions and pathways deregulated in ccRCC. We hypothesize that increased levels of ATP in connection with upregulated ATP exporters might lead to the activation of P2RY receptors, which in turn could inhibit the sodium transporter ENaC. Additionally, we see downregulation of ENaC subunits (SCNN1_A,B,D,G_), a phenomenon that is associated with hyponatremia and metastasis in ccRCC [44, 45, 49].

Using spatial transcriptomics data, we identified several disease-related factors in murine AKI using the modular and flexible LIANA+ CCC framework [27]. We inferred local interactions between spatially-adjacent variables, combined with non-negative matrix factorization, to identify an HNF4A signature absent in disease states. HNF4A was found to have a role in the AKI recovery of mice [52] and is known to bind to several lipids [51], which makes it an interesting target to investigate further.

Consequently, making use of diverse omics technologies, we demonstrate MetalinksDB’s utility not only in confirming interactions previously documented in the scientific literature but also in formulating hypotheses for novel ones.

The interactions described in the renal cancer use case were inferred using statistics representing metabolite or transcript deregulation in the bulk dataset. As such, they do not directly represent the deregulation of metabolite-protein binding. Moreover, the current approaches used to predict metabolite-receptor interactions from transcriptomics data [22, 24, 25], including the one used in this manuscript, are limited to the inference of metabolite abundances from gene expression, assuming a linear relationship between the two. Similarly, our approach to modeling metabolite presence assumes that enzymatic gene expression can serve as a proxy for metabolite abundance, neglecting the complex and largely non-linear nature of metabolite fluxes, which are influenced by the physicochemical state of the cells and their microenvironments, rather than merely the abundance of enzymes. Additionally, our modeling approach considers each metabolite as an independent entity, a simplification that could be addressed by adopting more sophisticated approaches [53–55] or by integrating multi-omics data, if available [56]. Thus, while here we infer putative metabolite-protein interactions, these remain only a hypothesis, to be validated.

As is commonly done in many biological databases, we had to decide which biological classes of protein-metabolite interactions to include and to make cutoffs in terms of a quality-coverage tradeoff. This tradeoff aims to strike a balance between incorporating potential interactions derived from structural predictions or text mining and the constraint of having only a few hundred manually curated interactions available. In line with this, we enable users to choose smaller, yet more confident, sets of interactions, such as those from literature curation, or alternatively, to investigate broader ranges of interactions. Further, MetalinksDB enables metabolite-receptor knowledge to be constrained to specific biological contexts, such as tissues, niches, and diseases. For example, in the case studies presented here, we constrained the interactions from MetalinksDB to metabolites that were previously reported in the kidney. Such a strategy is advantageous in two aspects: it potentially reduces erroneous predictions, while also improving the efficiency of computationally-demanding analyses. At the same time, since all prior knowledge databases have inherent limitations [5, 57], constraining interactions based on these annotations may inadvertently introduce biases.

Despite our efforts to enable flexible customization and to maintain interactions with high confidence, we acknowledge that MetalinksDB contains interactions that may not reflect a direct molecular binding event, but rather a link that exists through the close regulation of agents between metabolites and receptors. This is a consequence of how databases like STITCH, which comprises a significant portion of the interactions in MetalinksDB, are built. This highlights potential future directions, such as the inclusion of other generalistic metabolite-protein databases [58].

Moreover, the versatility of MetalinksDB, which facilitates straightforward access to multiple databases and filtering parameters, necessitates caution because the selection of resources may substantially influence CCC predictions [5].

Taken together, MetalinksDB provides a comprehensive and flexible resource for the growing field of metabolite-mediated CCC and will enhance data interpretation, particularly in studies where tissue context is of importance—as shown in the examples of kidney diseases. Moreover, experimental protocols that allow for a systematic characterization of the direct binding of proteins and metabolites [59, 60] will enable curation efforts in the future, for which MetalinkDB will be a suitable starting point. Beyond the use of cell–cell communication, MetalinksDB is set to streamline general metabolite-estimation tasks, enhancing emerging deep-learning and flux-based metabolic models [53, 54, 61]. We also anticipate that MetalinksDB will facilitate multi-modal data analyses, a particularly relevant utility with the technological advancements in single-cell and spatially-resolved metabolomics [62, 63], and in particular multi-omics [56].

## Methods

### Knowledge graph assembly

#### Interaction and annotation data

Information on metabolite protein-receptor interactions was obtained from STITCH [14], Rhea [16], NeuronChat [20], Cellinker [23], scConnect [24], and CellphoneDB [22]. After restricting the entries to human associations, the detailed interactions and actions in the STITCH datasets were obtained from the webpage (http://stitch.embl.de/). The ‘detailed interactions’ file as well as the ‘actions’ file were then loaded and subset to interactions having an annotated mode. Following this, the remaining interactions were cut down to only have the desired modes of action (e.g. activation, binding, and inhibition). The provided CIDs and Ensembl protein IDs were converted to HMDB [17] and UniProt IDs using the pypath module of Omnipath [9].

Directional information on which enzymes produce and degrade a metabolite was obtained from HMDB as well as genome-scale metabolic models [17–19]. HMDB protein and metabolite information was downloaded as a .xml file from the HMDB webpage (https://hmdb.ca/downloads) and parsed to a data frame using xml.sax and xml.etree. HMDB reaction information was scraped using the request and BeautifulSoup package (https://pypi.org/project/beautifulsoup4/). The HMDBP IDs obtained from scraping were translated to UniProt IDs, using the mappings obtained from HMDB protein data. In the current version of the HMDB adapter, the mapping links are pulled from Omnipath, which follows a similar parsing strategy to obtain the data.

The genome-scale metabolic models were downloaded from https://www.vmh.life/ [18] and https://github.com/SysBioChalmers/Human-GEM [19]. For the metabolic enzyme resource, we transformed the models, consisting of a stoichiometric matrix and information about the genes and metabolites, into a data frame consisting of gene-metabolite associations and a directionality. Associations resulting from reactions annotated as reversible were assigned both directionalities, except for proteins annotated as transporters. Missing identifiers were filled in by an ID translation table obtained from the HMDB metabolite data, as well as a table obtained from the metaboliteIDmapping R package (https://github.com/yigbt/metaboliteIDmapping).

Transporters were determined by the subsystem channel of the model, and a direction was defined using the compartment annotation. Transport from an organelle to the cytosol and from the cytosol to the extracellular milieu was assigned as outwards and inwards, respectively. Both association lists were then combined, and duplications were removed.

Further databases were leveraged to provide comprehensive annotations of metabolites and proteins. Uniprot data was downloaded via the API using the crossbar project (https://crossbar.kansil.org/project.php) BioCypher adapter. TransportDB2.0 and ‘Guide to Pharmacology’ data were obtained from the respective web pages (http://www.membranetransport.org/transportDB2/index.html, https://www.guidetopharmacology.org/download.jsp).

The code handling the above tasks used BioCypher adapters, which allow versioning and transparency of assembly. All the code used for the assembly of MetalinksDB is available at https://github.com/biocypher/metalinks. Executing the code yields several .csv files that can be loaded via Neo4j, which enables MetalinksDB to be queried using the cypher querying language [64].

During the knowledge graph assembly, several cutoffs are applied, which are summarized here for transparency. First, we excluded all interactions from STITCH that had no annotated interaction mode or a lack of HMDB and Uniprot identifiers. Second, we excluded all proteins that were not annotated as catalytic/nuclear receptors, GPRCs, ion channels, or transporters in the ‘IUPHAR Guide to Pharmacology’. We further included only interactions that had an interaction mode of ‘activation’, ‘inhibition’, or ‘binding’. In contrast to proteins known to act as receptors, interactions annotated as a ‘binding’ between a metabolite and an ion channel or between a metabolite and a transporter were ambiguous in most cases. For example, such binding events often depict generic cargo events, such as the intake of nutrients. Since we cannot distinguish the cargo events from events with signaling functions in the context of CCC—e.g. a metabolite blocks an ion channel—we have excluded such non-directed interactions by default. Nevertheless, these interactions are still accessible via custom cypher queries of the BioCypher knowledge graph.

We further applied a cutoff to the provided confidence level that comes with every STITCH connection. This cutoff was determined based on the distribution of manually curated interactions from CellphoneDB and NeuronChat (Supplementary Fig. S1). In brief, we investigated the STITCH confidence levels of interactions found in both the curated and STITCH data. The distribution gave us the impression that a substantial amount of true positives could be found, resulting in cutoffs of 200 for the database confidence score, 300 for the experiment score, 700 for the prediction score, and 900 for the combined one; the text mining was not taken into account. Finally, as our resource focuses on interactions of secreted metabolites (Supplementary Note S1), we excluded all metabolites that were not annotated as extracellular in the HMDB data.

This filtering strategy is a compromise from various priorities that other researchers may set differently. To address this problem, we build a web interface, where these filtering cutoffs can be adjusted to the user’s interest and are set by default to the thresholds matching the distributions of manually curated databases (Supplementary Fig. S1). Throughout this work, we use those thresholds, except for Section 2.4, where we use more stringent thresholds.

### Web interface

The web interface (https://metalinks.omnipathdb.org/) is based on the streamlit library (https://streamlit.io/) that uses the Neo4j driver to query data from the MetalinksDB knowledge graph. The code can be found here: https://github.com/saezlab/metalinks_web. The graph interface is built on the drugst.one html infrastructure [30].

### Data analysis

#### Application on bulk transcriptomics and metabolomics from ccRCC patient data

We performed differential abundance analysis using metabolomic and transcriptomic data from ccRCC patients. Specifically, we calculated differential statistics (moderated *t*-values and *P*-values) using limma [32] as a contrast between healthy versus tumor tissues—as done in Dugourd *et al.* 2021 [28]. We then joined these statistics with the metabolite-receptor interactions from MetalinksDB and calculated the mean of the *t*-values to obtain differential abundance summaries for each interaction. We used the false discovery rate method to correct for multiple testing. As a resource for metabolite-mediated CCC interactions, we used MetalinksDB after contextualizing it to metabolites found in the kidney, blood, or urine.

For the enrichment analysis, we downloaded the KEGG C2 set from MSigDB [65] (https://zenodo.org/records/10200150) and metabolic pathway annotations from a metabolic ccRCC atlas [33]. Then for each database, we estimated pathway enrichment scores using metabolite-transcript interaction *t*-values with Decoupler’s univariate linear model [66]. We assessed the robustness of these results by repeating the analysis with different cutoffs for the STITCH combined confidence score and ranking the interactions according to their absolute average *t*-values. Then, we divided the rank of every interaction by the total number of interactions above the threshold to get a relative rank from 0 to 1. For example, if there are 500 interactions above the threshold, the top-ranked interaction (rank 1) is divided by 500 (the total number of interactions above the threshold) to have a relative rank of 0.002. The lowest-ranking interaction with a rank of 500 would therefore get a 1. We used thresholds in the full range of 0–1000 with a step size of 20.

#### Application on kidney injury model spatial transcriptomics data

Five slides of AKI were obtained from a study by Dixon *et al.* [29]. Spots were log normalized, and genes were filtered for genes having more than 20 counts using the SCANPY package [67]. In line with recent developments, we used simple enrichment-like approaches to estimate the abundance of metabolites using the expression of their corresponding enzymes [20, 25]. Here, MetalinksDB was customized to only include metabolites found in the kidney and their corresponding receptors, with stricter cutoffs for the STITCH (>500 Database, >500 Experiment, and >900 combined score, urine as only biospecimen) to minimize the potential lower false positive rates. Using this customized version, we generated: (i) a consensus resource of manually curated metabolite-receptor interactions; (ii) sets of producing and degrading enzymes, respectively weighted as 1 and −1; and (iii) sets of transporters for each metabolite, with exporters being assigned to 1 and importers −1.

We then used a univariate linear regression model [66] to estimate metabolite abundances for each cell/spot. In a second step, inspired by NeuronChat [20] and scConnect [24], we calculated a transporter (export) score for each metabolite using a simple arithmetic mean, such that estimated metabolite abundances in each cell/spot, the export score of which is negative or 0, are set to 0. While we use a simple enrichment-like approach here, the metabolite estimation step with MetalinksDB and LIANA+ can be replaced by other more informed models [54], at the user’s discretion.

The inferred metabolite presence is then used to infer local ligand-receptor communication events using spatially-weighted cosine similarity [27]. Subsequently, we used a Gaussian radial kernel with a bandwidth of 100 to determine the spatial connectivities between spots. As a resource of metabolite-receptor interactions, we used a conservative set of connections mainly consisting of manually curated interactions. We set the negative metabolite values to 0 and calculated Cosine similarities between the inferred metabolite presence and corresponding receptors/transporters per slide. We then concatenated all the local scores from all slides and performed a non-negative matrix factorization with three factors, as determined by the elbow method according to the LIANA+ defaults.

To evaluate the robustness of our results, we generated several versions of the MetalinksDB resource using a range of STITCH combined score cutoffs from 0 to 950, increasing in increments of 50. For each version, we replicated the methodology described above, performing an NMF analysis to identify three factors. We then used Pearson correlation to the factor scores from each NMF run to determine the matching factors (patterns) relative to the original run. Subsequently, we calculated Pearson correlation coefficients again to compare the factor loadings of shared interactions across each run with those from the original one.

The graphical abstract as well as Figs 1, 3, 4, and S8 were created using Biorender (BioRender.com).

**Key Points**

- We present MetalinksDB, a comprehensive database comprising metabolite-protein interactions for cell–cell communication applications.
- MetalinksDB is easily accessible through a user-friendly webpage and can be customized to specific biological use cases.
- We use MetalinksDB on bulk multi-omics and spatial transcriptomics data to obtain mechanistic hypotheses in kidney diseases.

## Supplementary Material

FarrEtAl_metalinksDB_BiB_2024_Round3_Supp_corrected_order_bbae347

SuppTable03_Communication_scores_bbae347

## Data Availability

MetalinksDB Biocypher adapters are available via https://github.com/biocypher/metalinks with additional files at https://zenodo.org/records/10200150. The code used to generate the figures and analyses presented here is https://github.com/saezlab/MetalinksDB. MetalinksDB website is accessible via https://metalinks.omnipathdb.org/ and is built from https://github.com/saezlab/metalinks_web. We additionally provide programmatic access to MetalinksDB via LIANA+, along with detailed vignettes for their combined usage: https://liana-py.readthedocs.io/. Processed Renal Cell Carcinoma metabolome and transcriptome data [28] is available at https://github.com/saezlab/COSMOS_MSB/tree/main/data. Publicly available spatial transcriptomics data from acute kidney injury model mice [29] was obtained from the GEO repository (https://www.ncbi.nlm.nih.gov/geo/query/acc.cgi?acc=GSE182939).
