# Supplementary material for: MetalinksDB: a flexible and contextualizable resource of metabolite-protein interactions
Source: Brief Bioinform. 2024 Jul 22;25(4):bbae347. doi: 10.1093/bib/bbae347 (PMC11262834; doi:10.1093/bib/bbae347)
Supplement: FarrEtAl_metalinksDB_BiB_2024_Round3_Supp_corrected_order_bbae347 [file farretal_metalinksdb_bib_2024_round3_supp_corrected_order_bbae347.pdf]

Supplementary materials

Supplementary table 1. Databases for metabolite-mediated CCC

Name	Size# [Interactions]	Other contents	Curation	Availability	Source
**scConnect**	836	protein-protein interactions	Database	Open	GtP
**Cellinker**	341	protein-protein interactions	Databases	Open	GtP
**NeuronChat**	126†	protein-protein interactions	Manual	Open	
**CellphoneDBv5**	376	protein-protein interactions	Manual	Open	
**MEBOCOST**	421	**-**	Databases, Text-mining	Restricted*	

# The size reported reflects the number of interactions in each database with matching HMDB and UniProt IDs for metabolites and proteins, respectively.

* MEBOCOST authors explicitly forbid the usage of their resources outside of the MEBOCOST package.

† We report only human non-peptide interactions

GtP stands for ‘Guide to Pharmacology’

Supplementary table 2. Queries used in the manuscript and webpage

Cutoff	Database	Kidney multi-omics	Kidney spatial
**STITCH Experimental**	200	200	500
**STITCH Database**	300	300	500
**STITCH Prediction**	700	700	-
**STITCH Textmining**	-	-	-
**STITCH Combined**	900	900	900
**Cellular location**	Extracellular	Extracellular	Extracellular
**Tissue**	All	Kidney, All tissues#	Kidney, All tissues#
**Biospecimen**	All	Blood, Urine	Urine
**Interaction types**	Activation/Inhibition/ Binding*	Activation/Inhibition/ Binding*	Activation/Inhibition/ Binding
**Database version**	0.4.4	0.3.0	0.3.0
**Metabolite-receptor links**	10,242	3,863	2,667

Describes metabolites present in all tissues

* Excludes binding to transporters or ion channels (Methods)

Supplementary Note 1. *Metabolic signaling*

In the field of nutrient sensing and metabolite signaling, multiple, and sometimes conflicting, concepts of metabolic signaling exist [1,10,68]. This can impact the computation of metabolite-mediated CCC since these concepts influence what interactions are to be included in the prior knowledge. The diversity within the concepts may originate from the fact that most proteins can't surpass cell membranes, while metabolites, such as steroids, often do. They can because of their small size and chemical nature, which allows them to either diffuse through the membrane or use passive or active transport through the cell membranes [69]. Inside the cell, metabolites can interact with proteins in several ways: as substrates to metabolic enzymes, allosteric regulators, or scaffold molecules for protein complexes [59]. The allosteric regulation includes activation of proteins as in the case of nuclear receptors or inhibition due to binding to reactive sites.

For a cell, the consequences of a metabolite-protein interaction can range from very pronounced consequences, due to the activation of a signaling cascade triggered by a metabolite binding to a membrane or receptor, to minor consequences such as the metabolizing event or transport of a sugar or lipid that happens hundreds of times per second. While metabolizing or transport events are known to elicit strong cellular responses, most often through the signaling capacity of accumulating upstream or downstream metabolites [1,68], measuring such accumulation via transcriptomics is challenging. As such, to infer metabolite-mediated cell-cell communication from transcriptomics, we focus on primary messengers that directly elicit downstream signaling, thereby excluding transporters.

This strategy separates the different signaling modes from their downstream cascade and classifies them into the signal amplification capability of the direct target. As can be seen in **Supp. Figure S11**, as best examples of a clear primary messenger-signal amplification relationship **A** and **B** the signaling molecule binds to an outer membrane **-** or nuclear receptor. These receptors often have a clear downstream effect that already amplifies the signal, even if further signal integration may happen.

Similar to this, **C** describes first messengers that bind to ion channels or transporters that allow an in - or efflux of molecules that may have signaling properties (**Supp. Figure S11**). A more subtle interaction with a receiver cell is any interaction with an enzyme inside a cell that affects the enzyme's function (**E**). Finally, there are two classes of signaling interactions in which there is no defined metabolite-protein interaction: **D** which

illustrates the contact-dependent exchange of molecules between two cells, and **F** the change of a metabolic rate through the higher or lower abundance of a metabolite.

This definition of latter cases as signaling is of further relevance when considering including the results of several interactomics screens in the metabolite-receptor interactions list [59,60]. Since in their screens, a protein-metabolite interaction can stand for allosteric regulation of the protein, but also interactions as the substrate of the enzyme, we have to decide if we include the signaling events of the accumulation of upstream or downstream product of a reaction into account. In this manuscript, we decided to leave these cases out and therefore not include the interactomics screen results unless the interaction's nature is classified.

Another advantage of this strategy is that we can focus on certain cases, that for example incorporate a certain type of receptor and can make specific assumptions that ease the modeling for these cases. For instance, in the case of **A**, we don't need to consider importing transporters, while for **B, C, E,** and **F** we have to. Similarly, different prior knowledge resources are needed for the different cases that may change the structure of algorithms.

Supplementary Note 2. *Discussion of databases used*

A major source of information in the metabolite-receptor database is the STITCH database which serves as an extensive resource for over 20 million protein interactions, incorporating a diverse range of information from experimental data, databases, computational predictions, and text mining [17]. This comprehensive database provides crucial insights into the mode of binding, such as activation, inhibition, or binding, along with confidence scores for individual sources and combined analyses. Notably, the mode of binding is highly significant for downstream analysis; however, our understanding of what a binding event truly entails is mostly limited to activation events.

Similarly to Rhea [16], from which we include directed metabolite-enzyme sets in the MetalinksDB, Reactome [70] stores metabolite-enzyme interactions. However, Reactome is limited by the sparseness of directionality assignment or protein assignment for reactions, rendering it less useful for our purposes. However, the inclusion of reactions from Reactome in future versions should be easily done through the use of BioCypher adapters.

Despite the assigned confidence levels, determining the validity of a connection can be challenging, especially when limited information is available. Thus, MetalinksDB users are allowed to define their context-specific cutoffs based on relevant ground truth data. Neo4j, with its interactive graph representations and Cypher query language, presents an ideal tool for contextualizing protein interactions [64]. For this purpose, information from various secondary sources is utilized, predominantly HMDB, Recon3D, and UniProt, to obtain attributes of protein, metabolite, and association nodes. Nevertheless, the validity of these annotations for diseases, pathways, or tissue context is also under debate as in our previous investigations, we have observed ambiguity of annotations like the cellular location of proteins [5].

Our production-degradation resource (metabolic enzyme sets) is primarily based on the Recon3D metabolic model and the reaction webpages of the Human Metabolome Database (HMDB). Despite identifying some inaccuracies in the interactions, the Recon3D model remains widely accepted within the metabolomics field [71]. Each reaction on the Recon3D webpage is supported by evidence from the literature, enabling users to verify annotations. Another available model of human metabolism is the human HMR model, which was reported to provide better curated fatty acid metabolism [19,71].

HMDB sources most of its reactions from KEGG, another pathway database, though the latter's lack of open access presents challenges for direct usage [38]. Additionally, a significant portion of HMDB interactions are derived from BioTransformer, a model for predicting metabolism, such as lipid breakdown [72]. However, we approached these interactions with caution and frequently excluded them from our analysis.

Finally, the most straightforward inclusion choice for MetalinksDB were databases generated specifically in the context of CCC, including CellPhoneDB [22], NeuronChat [20], scConnect [24], and Cellinker [23]. Although these databases can be assumed to be of higher quality due to their manual curation efforts, they remain relatively limited in coverage.

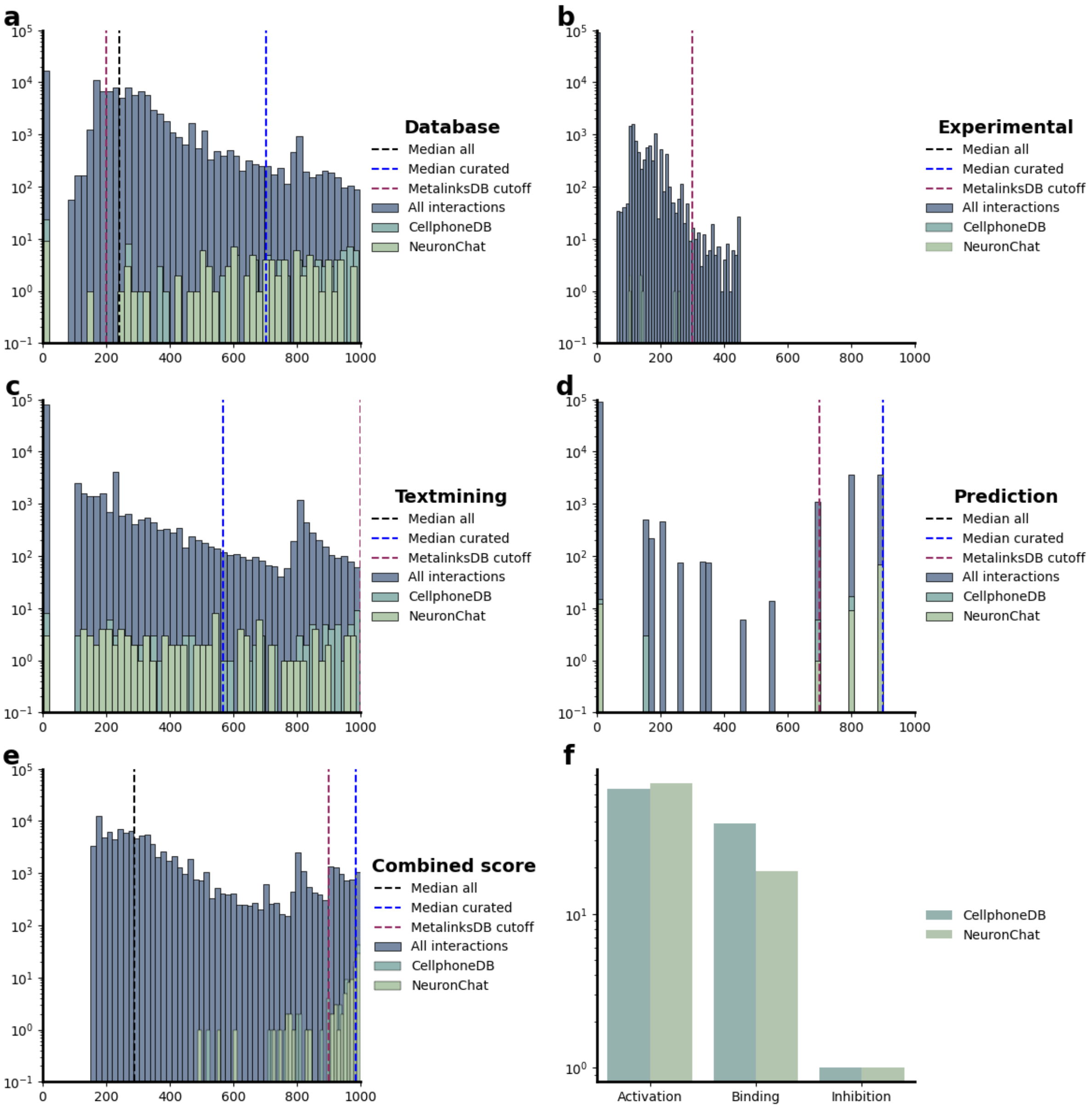

Supplementary Figure S1. *Comparison of STITCH confidence scores with manual curation.* ***A)*** *Database,* ***B)*** *Experimental,* ***C)*** *Textmining,* ***D)*** *Prediction, and* ***E)*** *Combined confidence scores of MetalinksDB interactions. Interactions that are also in manually curated databases (CellphoneDB, NeuronChat) are shown in green. Manually curated interactions have high Database, Prediction, and Combined scores, while few have experimental values and text mining appears evenly distributed.* ***F)*** *Histogram of interaction classification of CellphoneDB and NeuronChat, showing that most of their interactions are activating or binding.*

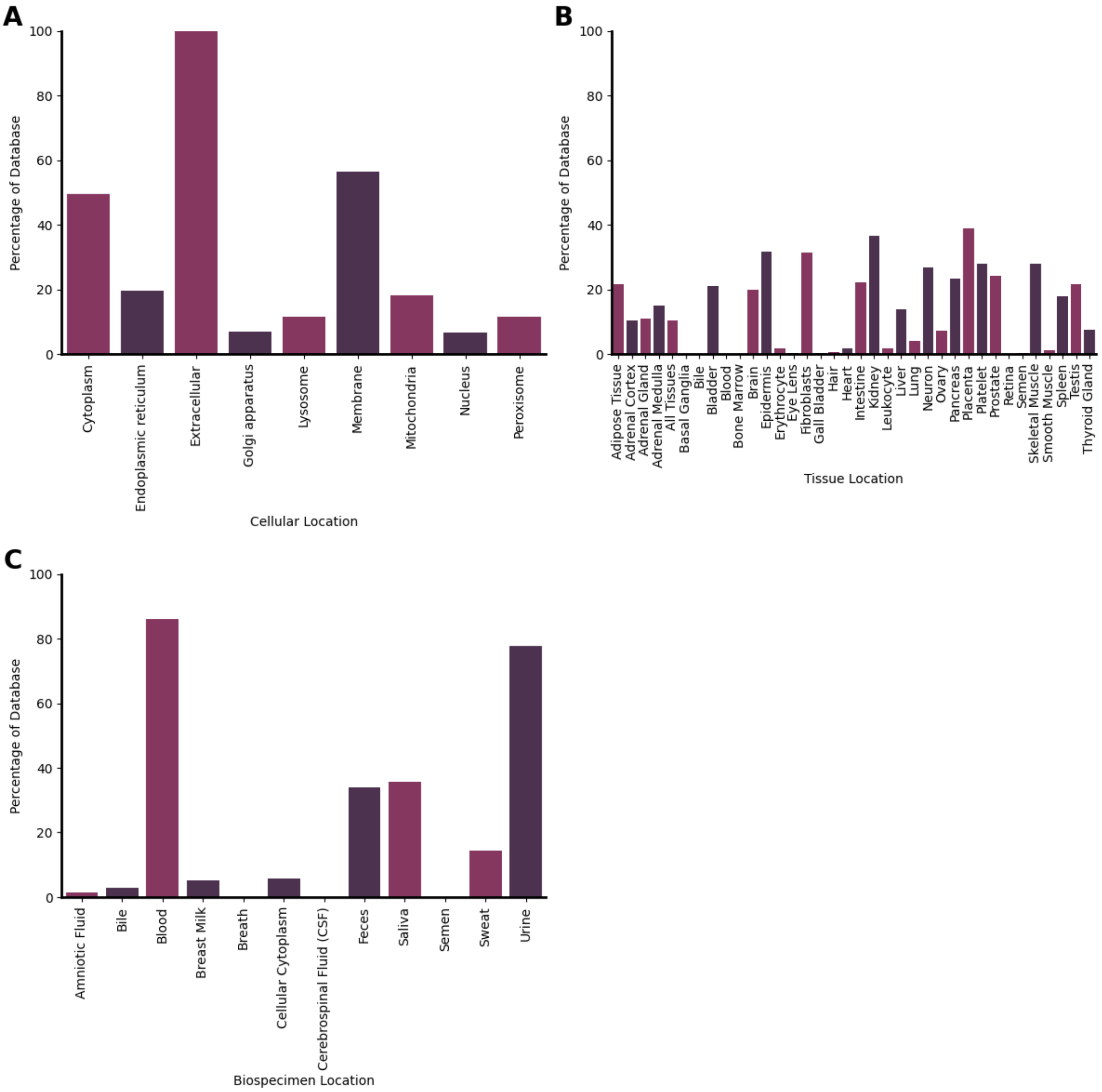

Supplementary Figure S2. *Fractions of metabolites in MetalinksDB belonging to annotation classes.* ***A)*** *Cellular locations of metabolites in MetalinksDB. All metabolites are annotated as extracellular, while approximately half of them are annotated as found in the membrane (0.57) or cytoplasm (0.50).* ***B)*** *Tissues in which metabolites of MetalinksDB are found. Metabolites are most commonly annotated as found in the placenta (0.39) followed by kidney (0.37) and epidermis (0.32). Many (13 out of 36) tissue annotations are prevalent in more than 20 percent of metabolites.* ***C)*** *Biospecimens locations of metabolites in MetalinksDB. Most metabolites in MetalinksDB can be found in the blood (0.86) or urine (0.78), while substantial amounts can also be found in saliva (0.36), feces (0.34), and sweat (0.14).*

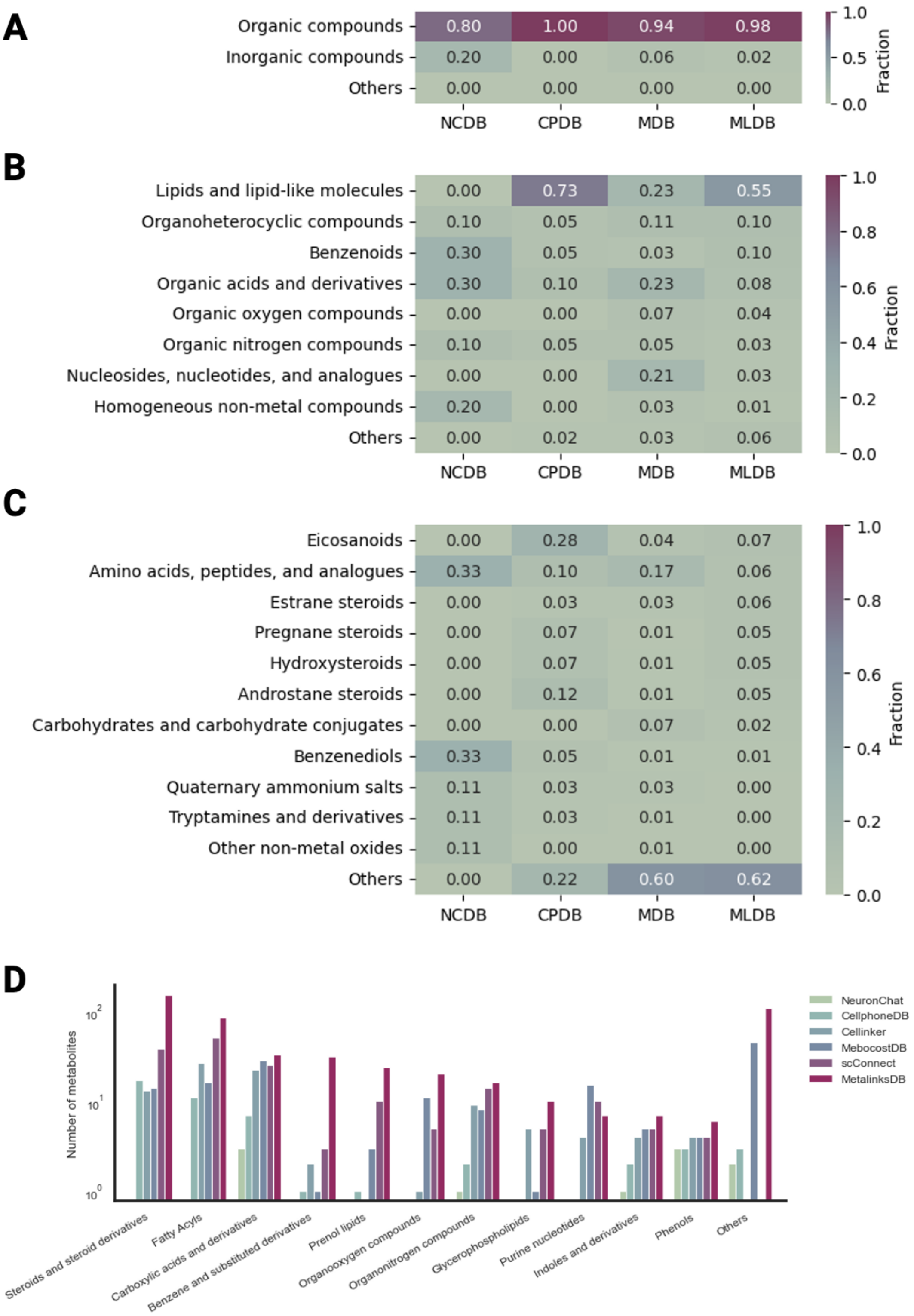

Supplementary Figure S3. *Metabolite class distributions throughout the databases.* ***A-C)*** *Heatmaps of fractions of metabolite classes, comparable to Figure 2B.* ***D)*** *Histogram of absolute values of metabolite classes.*

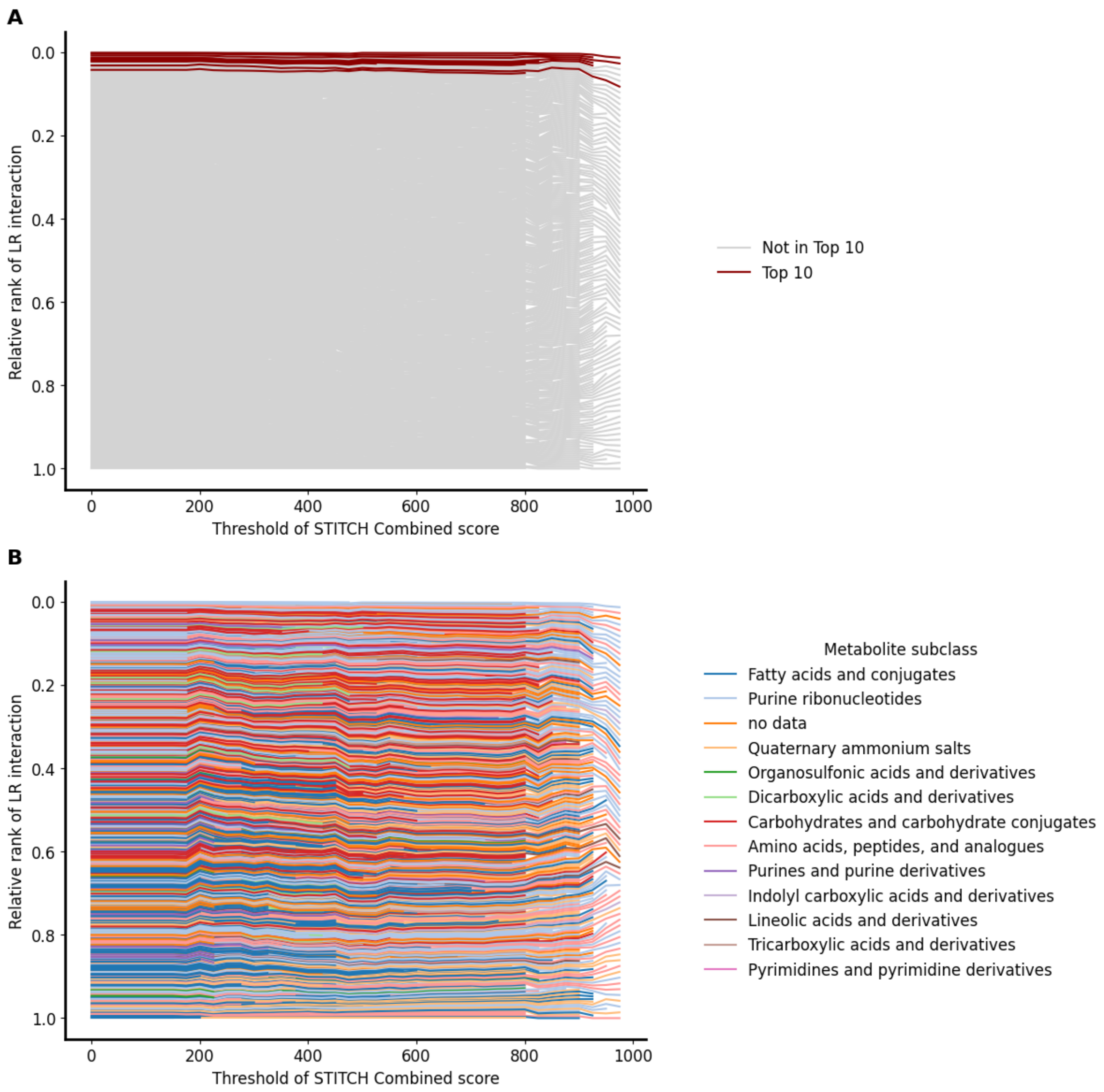

Supplementary Figure S4. *Normalized rank distributions (0-1) of interactions are robust across different ranges of STITCH confidence scores.* ***A)*** *Rank distributions of the top 10 interactions highlighted in Figure 3 (in red) against the remainder of the interactions (in grey). One line represents one ligand-receptor interaction.* ***B)*** *The distribution of metabolite classes in the metabolite ligands remains uniform with different STITCH combined confidence score cutoffs, suggesting that the different cutoffs don't bias the expected output metabolite classes (e. g. higher combined cutoff eliminates all interactions with purine ribonucleotides).*

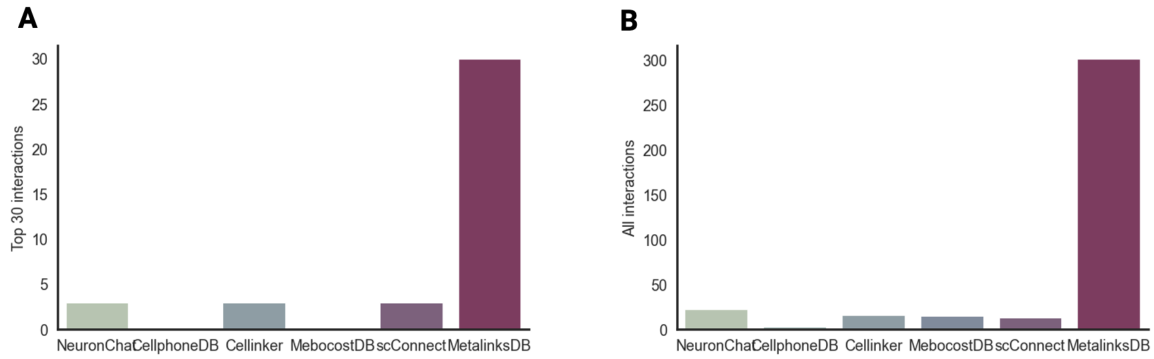

Supplementary Figure S5. *Kidney interactions prior knowledge found in other metabolite-receptor databases.* ***A)*** *In the top 30 ranked interactions, only up to 3 can be found in other databases.* ***B)*** *From all the interactions used in the analysis only a small fraction are also present in other databases (up to 20 compared to 290 in MetalinksDB).*

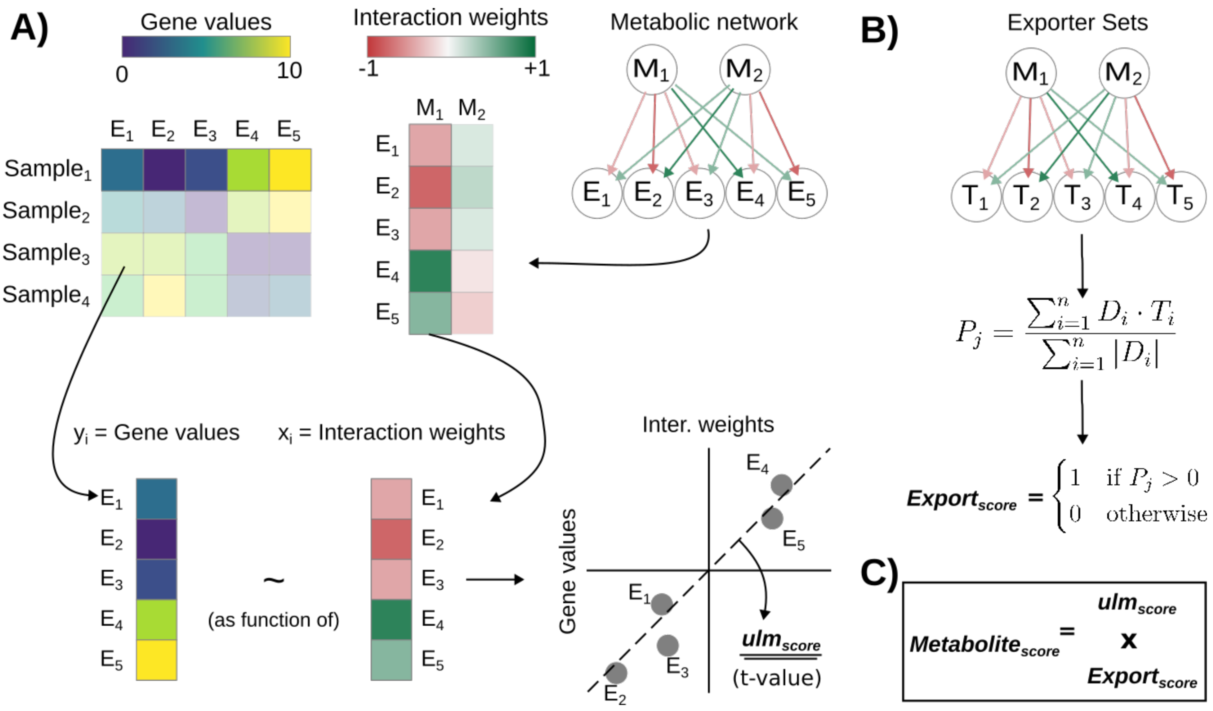

Supplementary Figure S6. *Metabolite presence estimation from gene expression data using MetalinksDB knowledge.* ***A)*** *Estimating Metabolite presence using MetalinksDB enzyme set knowledge with univariate linear regression from the decoupler-py package.* ***B)*** *Estimation of export score per metabolite using the weighted average of gene expression and transporter direction.* P_j *is the weighted export average score of metabolite* j. T_i *is the gene expression of transporter* i *and* D_i *is the direction for the transport of a given metabolite* j*, with* D_i *= +1 indicating that the transporter is involved in the export of the metabolite, while* D_i *= -1 corresponds to an import event.* ***C)*** *The calculation of a score combining prior knowledge of enzymes and transporters with gene expression to estimate metabolite presence from transcriptomics data.*

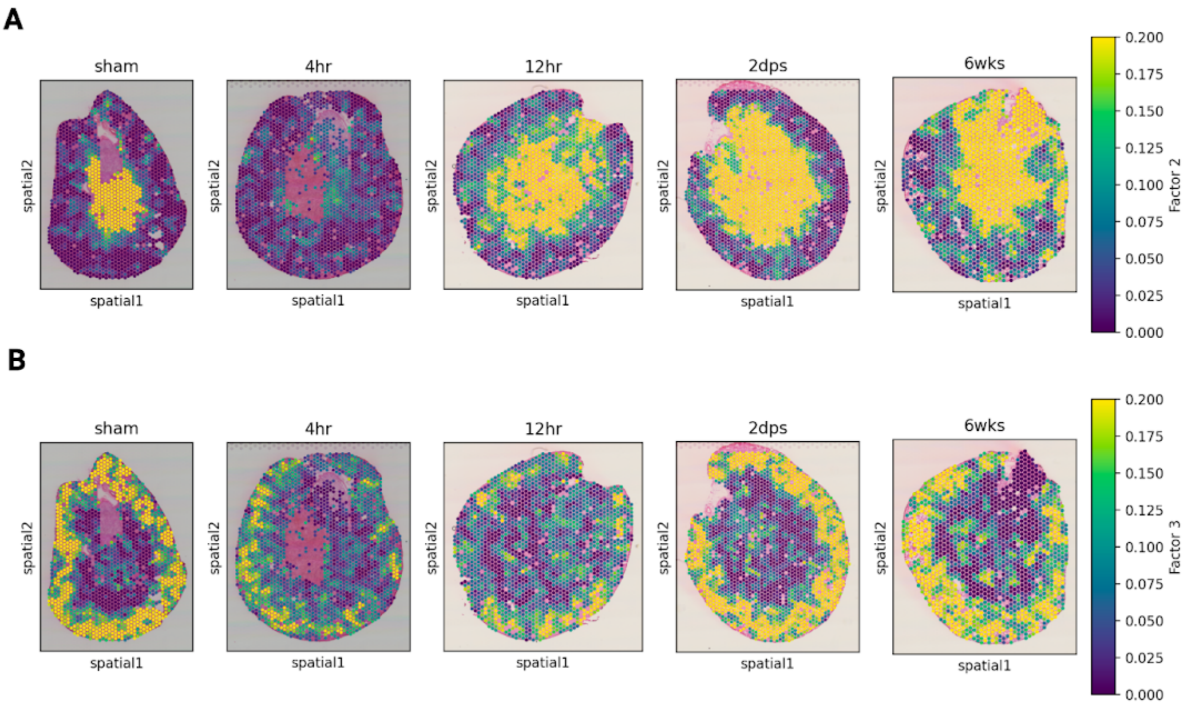

Supplementary Figure S7. *Factor scores from NMF analysis on communication scores from acute kidney injury data. Spatial distributions of Factors A) 2 and B) 3.*

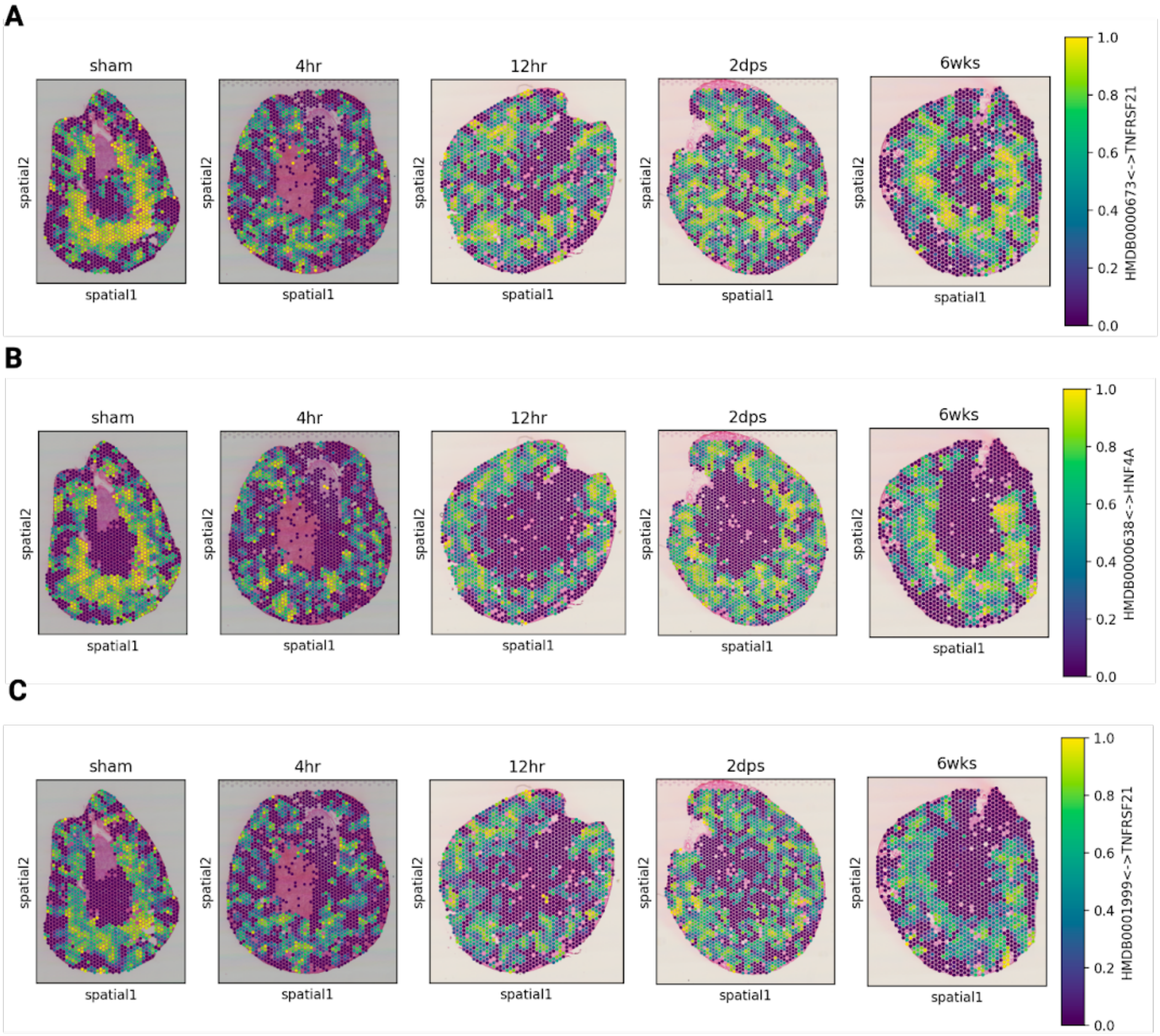

Supplementary Figure S8. *Factor loadings for the top interactions from NMF Factor 1.* ***A)*** *Interaction scores of Linoleic acid (HMDB0000673) with tumor necrosis factor receptor superfamily member 21 (TNFRSF21).* ***B)*** *Interaction scores of Dodecanoic acid (HMDB0000638) with HNF4A.* ***C)*** *Interaction scores of Eicosapentaenoic acid (HMDB0001999) with TNFRSF21.*

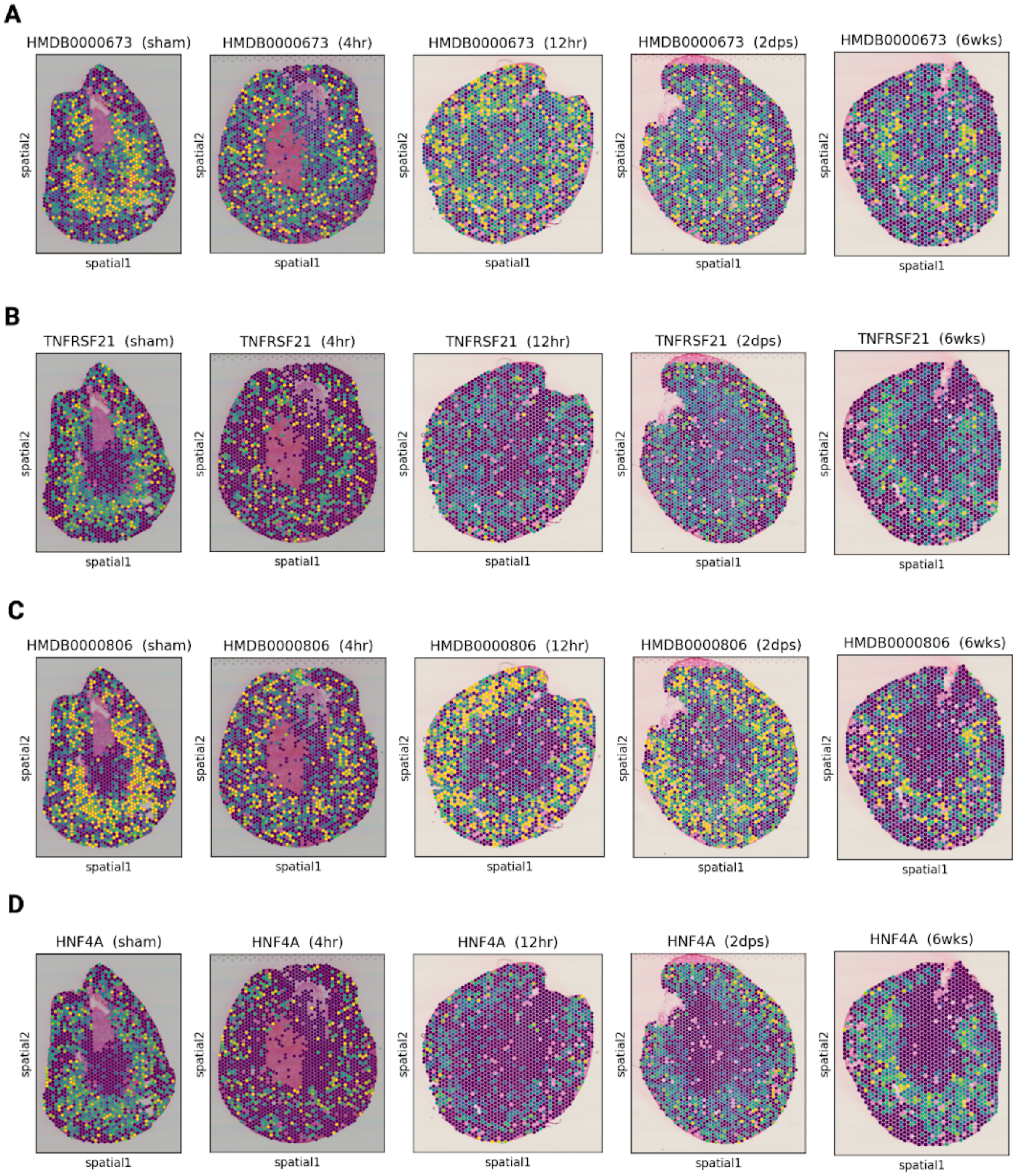

Supplementary Figure S9. *Spatial distributions of the components from interactions shown in* Supp. Figure S8*. Estimated abundances of **A)** linoleic acid (HMDB0000673) and **D)** myristic acid (HMDB0000806) as well as the expression of their corresponding receptors **B)** TNFRSF21 and **C)** HNF4A*.

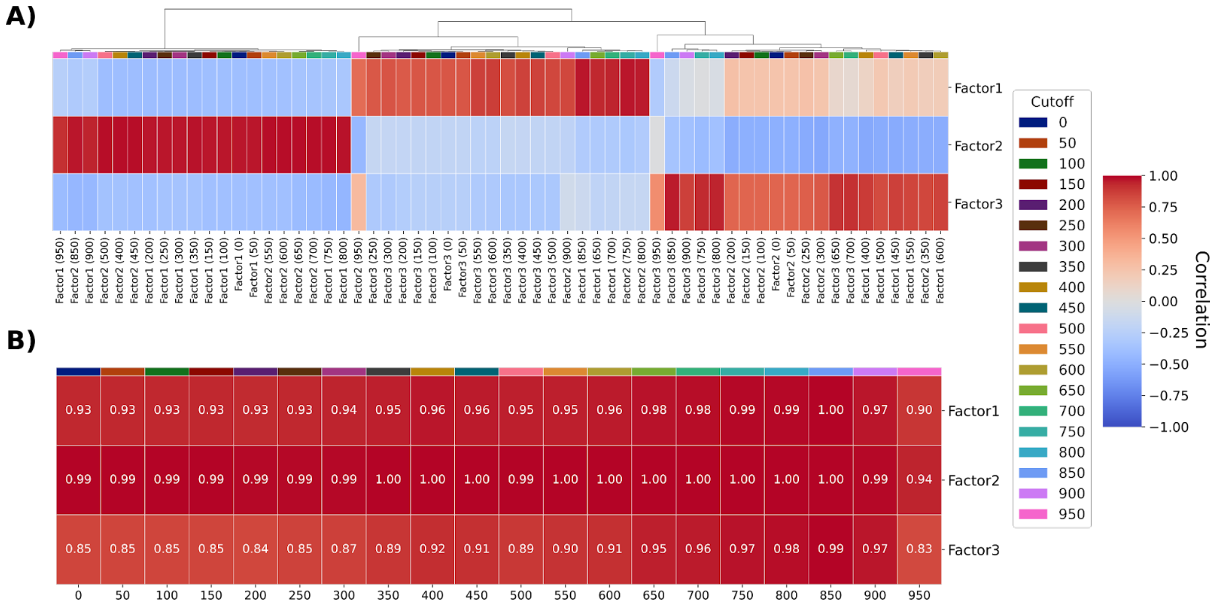

Supplementary Figure S10. *Robustness of intercellular communication patterns identified in spatial transcriptomics data using metabolite-receptor co-localisations and NMF.* ***A)*** *Pearson correlation of factor scores from various NMF runs, plotted across resource confidence (combined score) cutoffs ranging from 0 to 950.* ***B)*** *Pearson correlation of factor loadings from the 'original' run to their best-matching factors, determined by the correlation of factor scores in subpanel A, across different resource confidence cutoffs.*

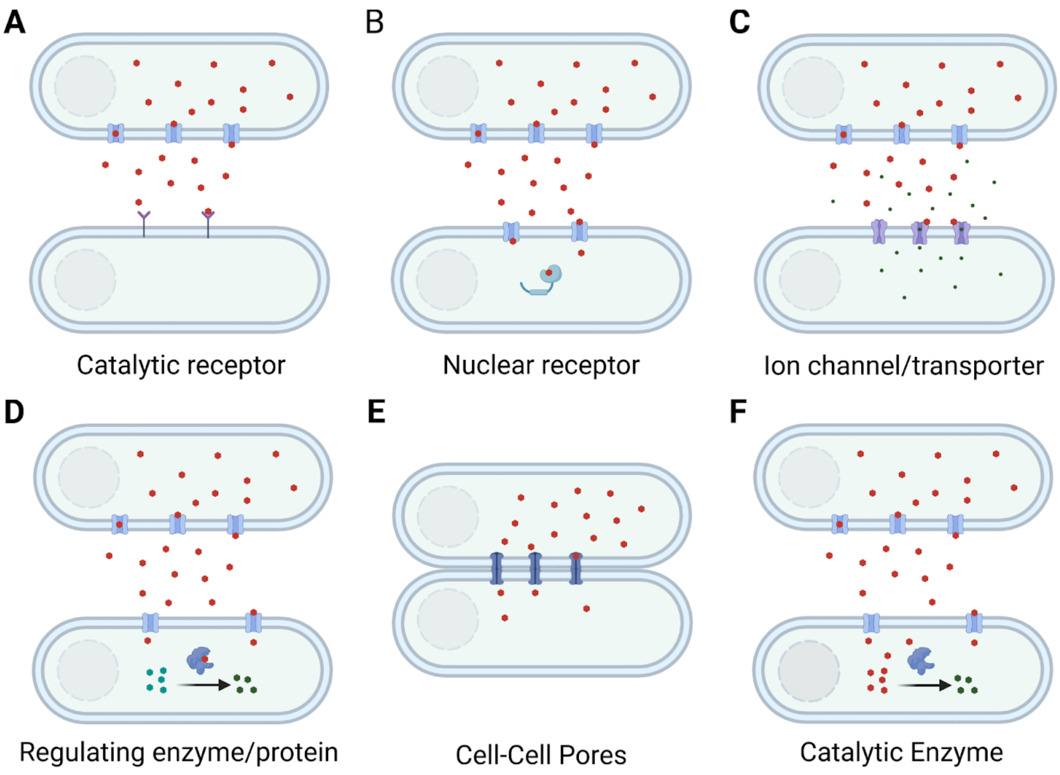

Supplementary Figure S11. *Modes of metabolite-mediated CCC. Metabolite-mediated CCC can be classified into several communication types. While some of the receptors are located in the membrane (**A & C**), intracellular receptors also exist (**B, D & F**). **E)** is a special case of signaling involving direct contact of cells through channels. All modes differ in the way molecule binding or exchange is amplified.*
